# *Macrophage Migration Inhibitory Factor* (MIF) Drives Murine Psoriasiform Dermatitis

**DOI:** 10.3389/fimmu.2018.02262

**Published:** 2018-10-02

**Authors:** Siegfried Bezdek, Lin Leng, Hauke Busch, Sadegh Mousavi, Dirk Rades, Markus Dahlke, Detlef Zillikens, Richard Bucala, Christian D. Sadik

**Affiliations:** ^1^Department of Dermatology, Allergy, and Venereology, University of Lübeck, Lübeck, Germany; ^2^Departments of Medicine and Pathology, Yale University School of Medicine, New Haven, CT, United States; ^3^Institute of Experimental Dermatology, University of Lübeck, Lübeck, Germany; ^4^Center for Research on Inflammation of the Skin (CRIS), University of Lübeck, Lübeck, Germany; ^5^Department of Radiation Oncology, University of Lübeck, Lübeck, Germany

**Keywords:** psoriasis, imiquimod-induced-psoriasiform dermatitis, IL-23-induced dermatitis, *Macrophage Migration Inhibitory Factor* (MIF), cytokines, chemokines, inflammation

## Abstract

The immunomodulator *Macrophage Migration Inhibitory Factor* (MIF) exerts pleiotropic immunomodulatory activities and has been implicated in the pathogenesis of diverse inflammatory diseases. Expression levels of MIF are also significantly elevated in the skin and serum of psoriasis patients, but the pathogenic significance of MIF in psoriasis is unknown. We have therefore addressed the role of MIF in two mouse models of psoriasis, namely in the imiquimod-induced psoriasiform dermatitis (IIPD) and the IL-23-induced dermatitis model. Daily treatment with Aldara™ cream, containing imiquimod, markedly increased the abundance of MIF in the skin and generated a cellular skin expression pattern of MIF closely resembling that in human plaque psoriasis. Deficiency in MIF significantly alleviated IIPD. On the clinical level, all hallmarks of psoriasiform dermatitis, including erythema, skin infiltration, and desquamation were reduced in *Mif*^−/−^ mice. On the histopathological level, MIF deficiency decreased keratinocyte hyperproliferation, inflammatory cell infiltration, specifically with respect to monocyte-derived cells, and dermal angiogenesis, suggesting that MIF may be involved in the pathogenesis of psoriasiform dermatitis through several mechanisms. Similarly, MIF deficiency also significantly reduced disease in the IL-23-induced dermatitis model, suggesting that MIF is involved in the pathogenic pathways activated by IL-23 and required to achieve full-blown psoriasiform dermatitis. Collectively, our results lend support to a possible disease-promoting role of MIF in psoriasis, which should be further investigated.

## Introduction

The immunomodulator *Macrophage Migration Inhibitory Factor* (MIF) is the earliest described lymphokine (1, 2). It is unique in its structure and biological activities, blending the properties of a cytokine, chemokine, and growth factor (3, 4). In the skin, MIF is constitutively expressed in the basal keratinocyte layer (5–8). MIF is a ligand of cell surface receptor complexes consisting of CD74 and CD44, CXCR2, CXCR4, or CXCR7 (9). It exerts pleiotropic, predominantly proinflammatory actions, such as T cell and macrophage activation, as well as chemoattraction of monocytes, neutrophils, and T cells (3, 10, 11).

MIF has been implicated in the pathogenesis of plaque psoriasis by observations that serum levels are elevated in psoriasis patients and that their PBMCs spontaneously release higher amounts of MIF than those of healthy controls (12). MIF expression is also induced in the epidermis and in the endothelium of dermal blood vessel in psoriatic plaques (6). Furthermore, polymorphisms in the promoter of the *MIF* gene are associated with a higher susceptibility to psoriasis (13). Despite this well-defined increased activity of MIF in psoriasis, the pathogenic role of MIF in this disease has not been investigated. Therefore, we have addressed the significance of MIF in the pathogenesis of psoriasis using two mouse models, the imiquimod-induced psoriasiform dermatitis (IIPD) and the IL-23-induced dermatitis model. In both models, skin inflammation clinically, histopathologically, and molecularly replicates major aspects of human plaque psoriasis (14–16). In the IIPD model, psoriasiform dermatitis is induced by topical application of Aldara™ cream, which contains imiquimod, an agonist of TLR7 and antagonist of adenosine receptors, as well as isostearic acid, an activator of the NLRP1 inflammasome, as pharmacologically active compounds (16). The IIPD model was developed upon the observation that Aldara™, as a side effect, can elicit or exacerbate psoriasiform dermatitis in psoriasis patients or psoriasis-prone individuals (15). IIPD is driven by several parallel and partially redundant pathways directly activated by Aldara™. Among these pathways, the IL-23/IL-17 pathway is most important, but also type I interferons, IL-1α/β, and TNF-α contribute to achieve full-blown dermatitis (17–20).

The IL-23-induced dermatitis model is based on intradermal injections of recombinant IL-23 (14), which establishes a gene expression pattern in the skin resembling that in human psoriatic skin lesions (21). The IL-23/IL-17 pathway is also a most critical pathway for human plaque psoriasis. Its inhibition is therapeutically exploited and achieves most significant clinical benefits (22).

In this study, we show that MIF is highly expressed in psoriasiform skin lesions in both the IIPD and the IL-23-induced dermatitis model. Herein, it exhibits a cellular expression pattern closely resembling that in human plaque psoriasis. Deficiency in MIF significantly blunts psoriasiform dermatitis in both models, suggesting that MIF possibly acts as effector molecule downstream of IL-23. Our more detailed investigation reveals that MIF is involved in orchestrating the recruitment of monocytes into the dermis, which has lately been highlighted as crucial for the emergence of psoriasiform dermatitis in both models (23).

## Materials and methods

### Mice

*Mif* ^−/−^ mice on the *C57BL/6* background, described before (24), and *C57BL/6* wild-type mice were bred in our animal facility at the University of Lübeck. Mice were used for experiments in age- and sex-matched groups at the age of 8–10 weeks. All animal experiments had been approved by the state government of Schleswig-Holstein.

### Performance of the imiquimod-induced psoriasiform dermatitis (IIPD) mouse model

For IIPD on the back skin, a 2 × 3 cm area was depilated 2 days before the first application of 50 mg Aldara™ cream (Meda, Solnau, Sweden), containing 5% imiquimod, daily on this area for five consecutive days, as previously described (25, 26). Dermatitis was evaluated using a modification of the *Psoriasis Activity and Severity Index* (PASI): erythema, infiltration, and desquamation were individually scored on a scale from 0 to 4 with 0, none; 1, mild; 2, moderate; 3, marked; 4; severe. The scores of these individual aspects of dermatitis were summed up to obtain the cumulative score. At the dorsal ear skin, IIPD was induced by topical application of 5 mg Aldara™ cream for 5 consecutive days. The dorsal-ventral distance of the ear was measured daily before the application of Aldara™ using a micrometer (Mitutoyo Europe, Neuss, Germany). To determine ear swelling, the dorsal-ventral distance measured on day 0 was subtracted from these values. At the end of the experiments, mice were euthanized by heart puncture and serum was harvested and stored at −20 °C until usage.

### Histopathology

For histopathology, skin biopsies were fixed in 4% Histofix® solution (Carl Roth, Karlsruhe, Germany), embedded in paraffin, and cut into 6 μm sections. For histopathology, sections were subsequently H&E stained. The epidermal thickness was assessed by measuring the distance between the dermal-epidermal junction and the epidermal surface using BZ-II Analyzer software (Keyence, Neu-Isenburg, Germany).

### Mif inhibition by (ISO-1)

1 mg 4,5-Dihydro-3-(4-hydroxyphenyl)-5-isoxazoleacetic acid methyl ester (ISO-1), purchased from Santa Cruz Biotechnology (Santa Cruz, CA, USA) and solved in 10% DMSO in H_2_O, were injected i.p. in a volume of 150 μl daily starting 2 days before the first application of Aldara™. The control group received only the vehicle.

### Immunofluorescence (IF) stainings

For IF stainings, skin biopsies were embedded in Tissue-Tek® Cryomold® (VWR, Darmstadt, Germany) before 6-μm sections were cut and stored at −20°C until usage. For IF staining of MIF, anti-MIF polyclonal rabbit antibodies were generated, isolated, and used for staining, as previously described (27), using donkey anti-rabbit IgG labeled with hexafluor 594 as secondary antibody. To distinguish MIF expression in endothelial cells, dermal blood vessels were identified in brightfield capture overlays. All other antibodies used for IF in this study were commercially available and are listed in Table S2. They were used according to the manufacturers' instructions. After staining, slides were mounted with DAPI fluoromount G (SouthernBiotech, Melbourne, Australia). IF stainings were visualized and photographed using the BZ-9000E series Keyence microscope and BZ-II Analyzer software (Keyence GmbH, Neu-Isenburg, Germany). The number of Ki-67^+^ keratinocytes per μm^2^ in the epidermis was quantified by BZ-II Analyzer software (Keyence, Neu-Isenburg, Germany). Using BZ-II Analyzer software (Keyence GmbH, Neu-Isenburg, Germany), the extent of dermal infiltration by F4/80- and CD68-positive cells was quantified by determining the percentage of the F4/80- and CD68-positive area per high power field (HPF), and angiogenesis was quantified by determining the number of CD31^+^ vessels in the dermis per HPF.

### Isolation of RNA and expression analysis

Total RNA was extracted from skin biopsies using TRIzol® reagent (Thermo Fischer Scientific, Waltham, MA, USA) according to the manufacturer's instructions. RNA concentrations were measured by Nanodrop 2000c spectrophotometer (Thermo Fischer Scientific, Waltham, MA, USA). Transcription of 100 ng of total RNA using the ReverseAid **First** Strand cDNA Synthesis Kit (Thermo Fischer Scientific, Waltham, MA, USA) and subsequent qPCR using the SYBR Select Master Mix (Thermo Fischer Scientific, Waltham, MA, USA) were performed according to the manufacturers' instructions. Primers were purchased from biomers.net (biomers.net GmbH, Ulm, Germany), and their sequences are listed in Table S1. qPCR was run on the Eppendorf Mastercycler ep Realplex (Eppendorf, Hamburg, Germany). The cycling conditions were 50°C for 2 min, 95°C for 2 min, followed by 40 cycles each of 95°C for 15 sec, and 60°C for 1 min each. The expression level of the gene of interest was normalized to the GAPDH mRNA expression level.

### Determination of IL-17A and MIF serum levels

Serum levels of IL-17A and MIF were determined by ELISA using kits from BioLegend (San Diego, CA, USA) and R&D Systems (Minneapolis, MN, USA), respectively, according to the manufacturers' instructions.

### Induction of recombinant IL-23-induced dermatitis

IL-23-induced dermatitis was induced and evaluated, as previously described (14). Briefly, 0.5 μg of recombinant murine IL-23 (eBioscience, Frankfurt, Germany), solved in 1% BSA in PBS, was injected i.d. in a volume of 20 μl into the dorsal surface of right ear, vehicle control (1% BSA in PBS) into the left ear of recipient mice every other day, starting day 0 of the experiment. Subsequently, ear swelling was determined, as described above.

#### Generation of bone marrow chimera

Bone marrow chimera were generated, as described previously (28). Briefly, recipient mice were irradiated (10 Gy, 10 min) and afterwards i.v. injected with 10^7^ bone marrow cells, freshly isolated from the femores and tibiae of donor mice. Eight weeks after reconstitution, chimeric mice were checked for chimerism and used for experiments.

### Statistics

Clinical disease scores and ear swelling were analyzed by two-way-ANOVA with Holm-Sidak's multiple comparison test. Epidermal thickness, protein expression levels in histopathology stainings, and qPCR results were first tested for normality by Shapiro-Wilk normality test. Normally distributed data were tested for statistical significance by unpaired *t*-test or one-way ANOVA with Bonferroni's multiple comparison test, if two or more groups were compared. Statistical significance of datasets not proven to be normally distributed was evaluated by Kruskal-Wallis test with Dunn's multiple comparison test. The respective test applied is indicated in the figure legends. *p* < 0.05 was considered statistically significant. All calculations were performed with GraphPad Prism 7.0c (GraphPad Inc., San Diego, CA, USA). All data are presented as mean ± SEM.

## Results

### Abrogation of MIF ameliorates IIPD

**Figure 1:**
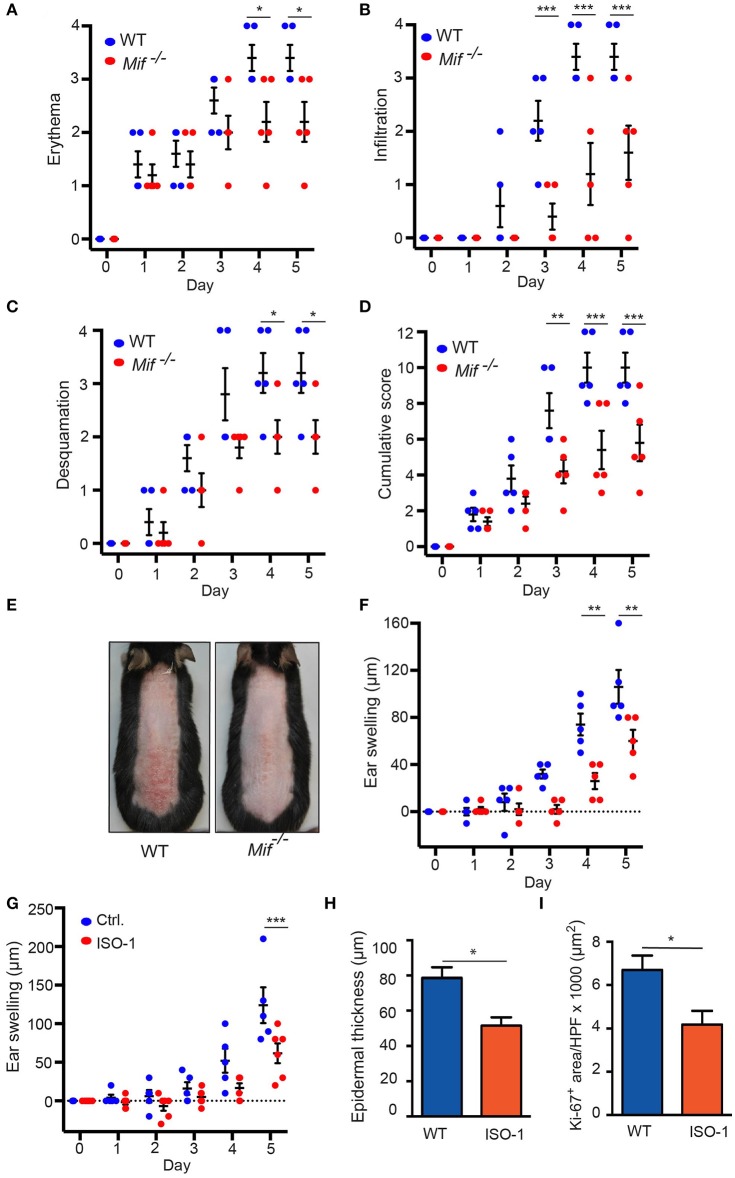
IIPD is attenuated in *Mif*^−/−^ mice. Clinical course of IIPD on the dorsal skin of WT and *Mif* ^−/−^ mice evaluated by **(A)** erythema, **(B)** infiltration, **(C)** desquamation, and **(D)** their cumulative score. **(E)** Representative clinical presentation of the dorsal skin on day 5. **(F)** Time course of ear swelling (μm). **(G)** Time course of ear swelling, **(H)** epidermal thickness, and **(I)** Ki-67 protein expression levels in Aldara™ treated ear skin in ISO-1 or vehicle treated mice on day 5. Results in **(A–D, F, G)** were analyzed by two-way ANOVA and Holm-Sidak's multiple comparison test, results in **(H, I)** by unpaired *t*-test. ^*^p < 0.05, ^**^p < 0.01, and ^***^p < 0.001 comparing WT to *Mif*^−/−^ mice (*n* = 5 mice/group). One representative of two independent experiments is shown.

To elucidate the role of MIF in murine psoriasiform dermatitis, we first compared skin inflammation in wild-type and *Mif* ^−/−^ mice in the IIPD mouse model. Application of Aldara ™ to the skin of the back or the dorsal ear elicited psoriasiform skin inflammation, including erythema, skin infiltration, desquamation, and swelling of the ear in both wild-type and *Mif*^−/−^ mice within 2–3 days. The severity of psoriasiform dermatitis however, was significantly attenuated in *Mif*^−/−^ mice compared to wild-type mice. Thus, erythema, skin infiltration, and desquamation were significantly reduced in *Mif*^−/−^ mice (Figures 1A–C). In sum, these differences in the single clinical assessment criteria of psoriasiform dermatitis resulted in a significant decrease of the cumulative disease score by ~40% in *Mif* ^−/−^ mice compared to wild-type mice at the end of the experiment on day 6 (Figures 1D,E). Additionally, ear swelling, another parameter for the severity of skin inflammation induced by application of Aldara™ onto the dorsal ear skin, was attenuated in *Mif*^−/−^ mice (Figure 1F). Next, we evaluated the effect of pharmacological inhibition of MIF by the MIF inhibitor ISO-1 (29) on psoriasiform dermatitis. For this purpose, wild-type mice were treated with 1 mg ISO-1 i.p. daily starting 2 days before the first application of Aldara™ onto the dorsal ear skin. ISO-1 alleviated psoriasiform dermatitis assessed by ear swelling, epidermal hyperproliferation, and epidermal thickness (Figures 1G–I).

### Cellular dynamics of psoriasiform dermatitis in wild-type *vs. mif^−/−^* mice

**Figure 2:**
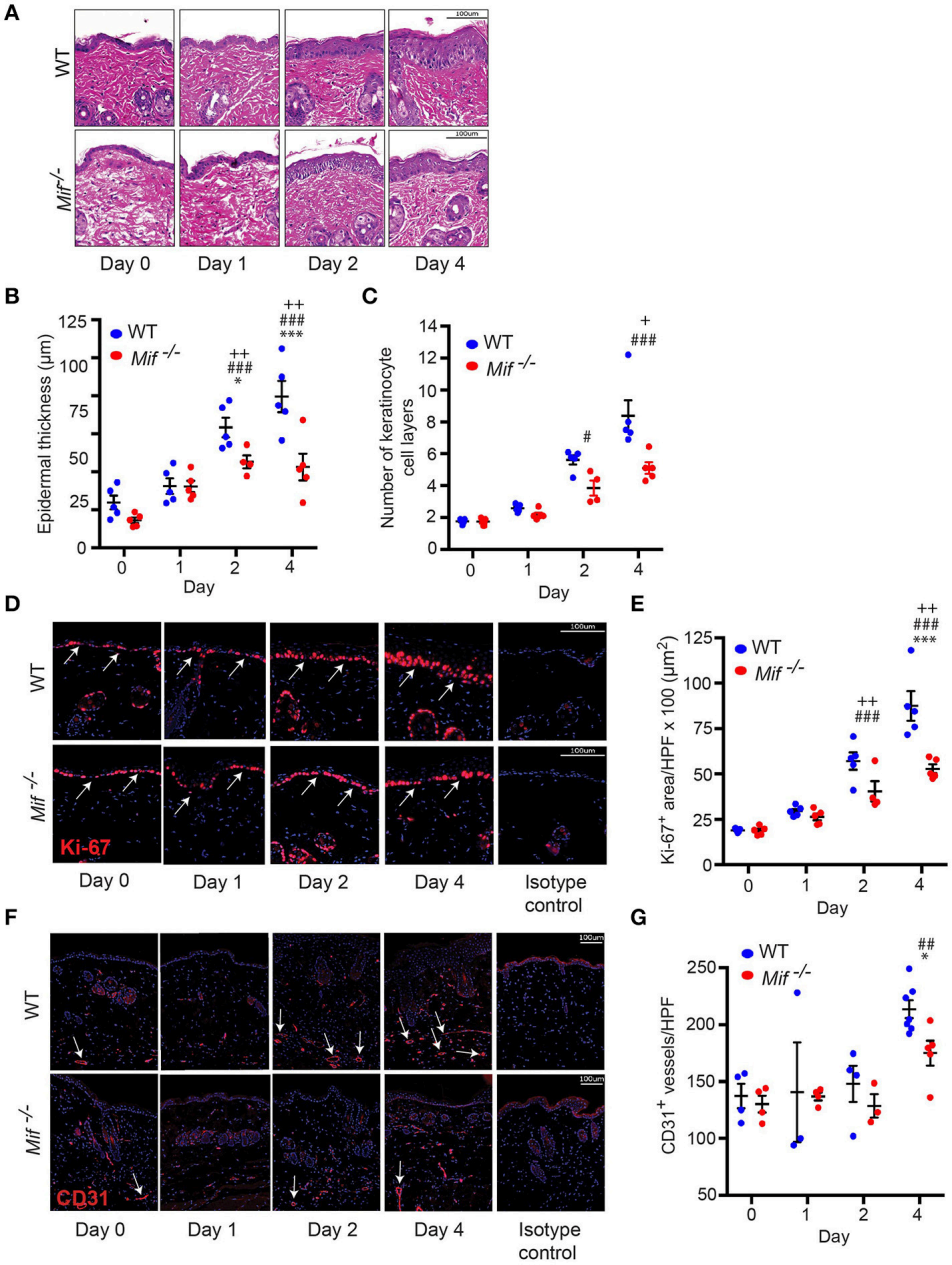
MIF drives epidermal hyperplasia and angiogenesis in psoriasiform dermatitis. The progression of histopathological changes in Aldara™ treated dorsal skin was assessed in WT and *Mif* ^−/−^ mice over time. **(A)** Representative H&E stainings, time course of **(B)** epidermal thickness, **(C)** the number of keratinocyte cell layers. **(D)** Ki-67 immunofluorescence staining and **(E**) Ki-67 protein expression levels in the epidermis. **(F)** CD31 immunofluorescence and **(G)** number of CD31^+^ vessels/HPF. Results in **(B, E, G)** were tested for statistical significance by one-way ANOVA and Bonferroni's multiple comparison test, results in **(C)** were tested by Kruskal-Willis test. ^*^p < 0.05; ^***^p < 0.001 WT *vs. Mif* ^−/−^ mice; ^##^*p* < 0.01; ^###^*p* < 0.001 WT mice day 0 vs. WT mice day indicated; ^+^, *p* < 0.05; ^++^, *p* < 0.01 *Mif* ^−/−^ mice day 0 *vs. Mif* ^−/−^ mice day indicated (*n* = 3–8 mice/time point/group). Scale bars represent 100 μm. Arrows in **(D)** indicate Ki-67 expression, in arrows in **(F)** indicate CD31 expression. One representative of two independent experiments is shown.

To gain insight into the dynamics of psoriasiform dermatitis in wild-type and *Mif*^−/−^ mice, we profiled the histopathological alterations of the skin over time. For this purpose, IIPD was induced in wild-type and *Mif*^−/−^ mice. Mice were euthanized prior to the first application of Aldara ™ on day 0 as well as on days 1, 2, and 4. Histopathology of the back skin of these mice revealed that while alterations typical of psoriasiform dermatitis, including immune cell infiltration of the dermis, keratinocyte hyperproliferation, and neoangiogenesis, were evident in both groups, they were all diminished in *Mif*^−/−^ mice (Figure 2A). Thus, quantification of keratinocyte hyperproliferation by determining epidermal thickening (Figure 2B), the number of keratinocyte cell layers (Figure 2C), and the epidermal expression of the proliferation marker Ki-67 (Figures 2D,E) revealed that keratinocyte hyperproliferation gradually increased in both wild-type and in *Mif*^−/−^ mice over the observation time of 4 days, however, each of these parameters in *Mif*^−/−^ mice reached a level of approximately only 50% of that in wild-type mice. Dermal angiogenesis was assessed by determining the expression of the endothelial lineage marker CD31 in the dermis. By day 4, significant angiogenesis proceeded in the dermis of wild-type mice, but remained only at a moderate level in *Mif*^−/−^ mice (Figures 2F,G).

**Figure 3:**
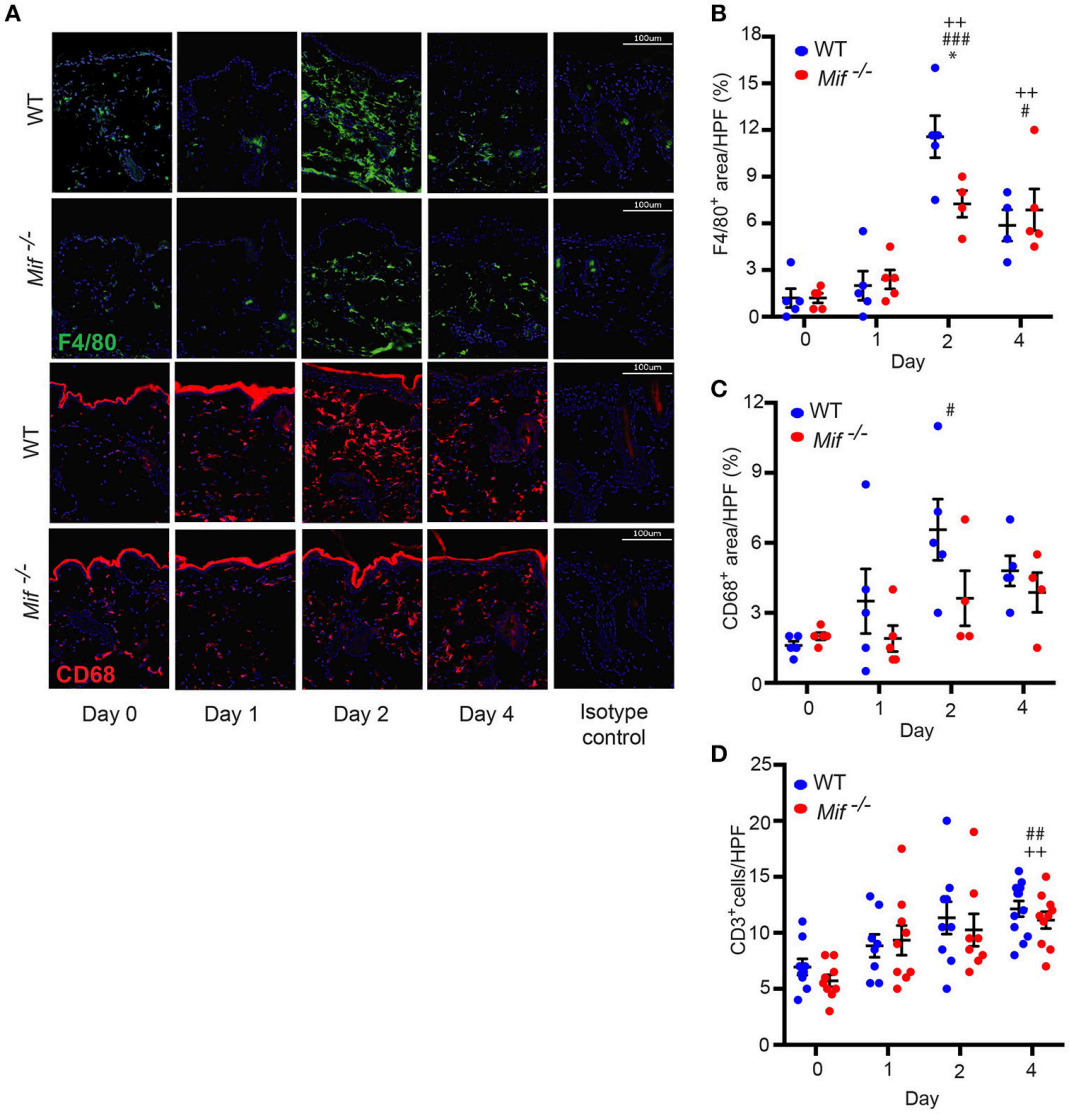
MIF facilitates monocyte recruitment into the dermis in IIPD. Time course of F4/80 and CD68 positive cell infiltration in IIPD. **(A)** Representative immunofluorescence stainings for F4/80 and CD68 positive cell infiltration in WT and *Mif* ^−/−^ mice on days 0 through 4 in Aldara™ treated skin. Respective quantification of **(B)** F4/80^+^ and **(C)** CD68^+^ areas/HPF. **(D)** Number of CD3^+^ cells/HPF in the dermis on days 0 through 4. Results in **(B)** and **(D)** were tested for statistical significance by one-way ANOVA and Bonferroni's multiple comparison test, results in **(C)** by Kruskal-Wallis test. ^*^, *p* < 0.05 WT *vs. Mif* ^−/−^ mice; ^#^, *p* < 0.05; ^##^, *p* < 0.01; ^###^, *p* < 0.001 WT mice day 0 *vs*. WT mice day indicated; ^++^, *p* < 0.01 *Mif* ^−/−^ mice day 0 *vs. Mif* ^−/−^ mice day indicated (*n* = 5–12 mice/time point/group). Scale bars represent 100 μm. One representative of two independent experiments is shown.

The recruitment of T cells, monocytes/macrophages, and neutrophils into the skin is a hallmark of psoriasiform dermatitis (23). We therefore profiled the temporal dynamics of T cell, monocytes/macrophage, and neutrophil recruitment into the skin in wild-type *vs. Mif*^−/−^ mice. Prior to Aldara™ administration, there was no difference in the cellular composition of the skin in wild-type and *Mif*^−/−^ mice. Upon Aldara™ treatment, the dermis of wild-type mice became densely infiltrated by monocyte/macrophages (Figure 3A), which were identified by their expression of monocyte/macrophage surface markers F4/80 and CD68 (30). The recruitment of monocytes/macrophages was reduced in *Mif*^−/−^ mice. This difference between wild-type and *Mif*^−/−^ mice was most pronounced at the peak of F4/80^+^ and CD68^+^ cell infiltration on day 2 (Figures 3B,C). T cells were identified by CD3 expression. They were recruited into the dermis of both wild-type and *Mif*^−/−^ mice upon Aldara™ without a difference between the two strains (Figure 3D). Neutrophils, identified by Ly-6G expression, were recruited into the dermis only in small numbers with no detectable difference between the two strains (results not shown).

### Aldara™ induces the expression of MIF in resident skin cells

**Figure 4:**
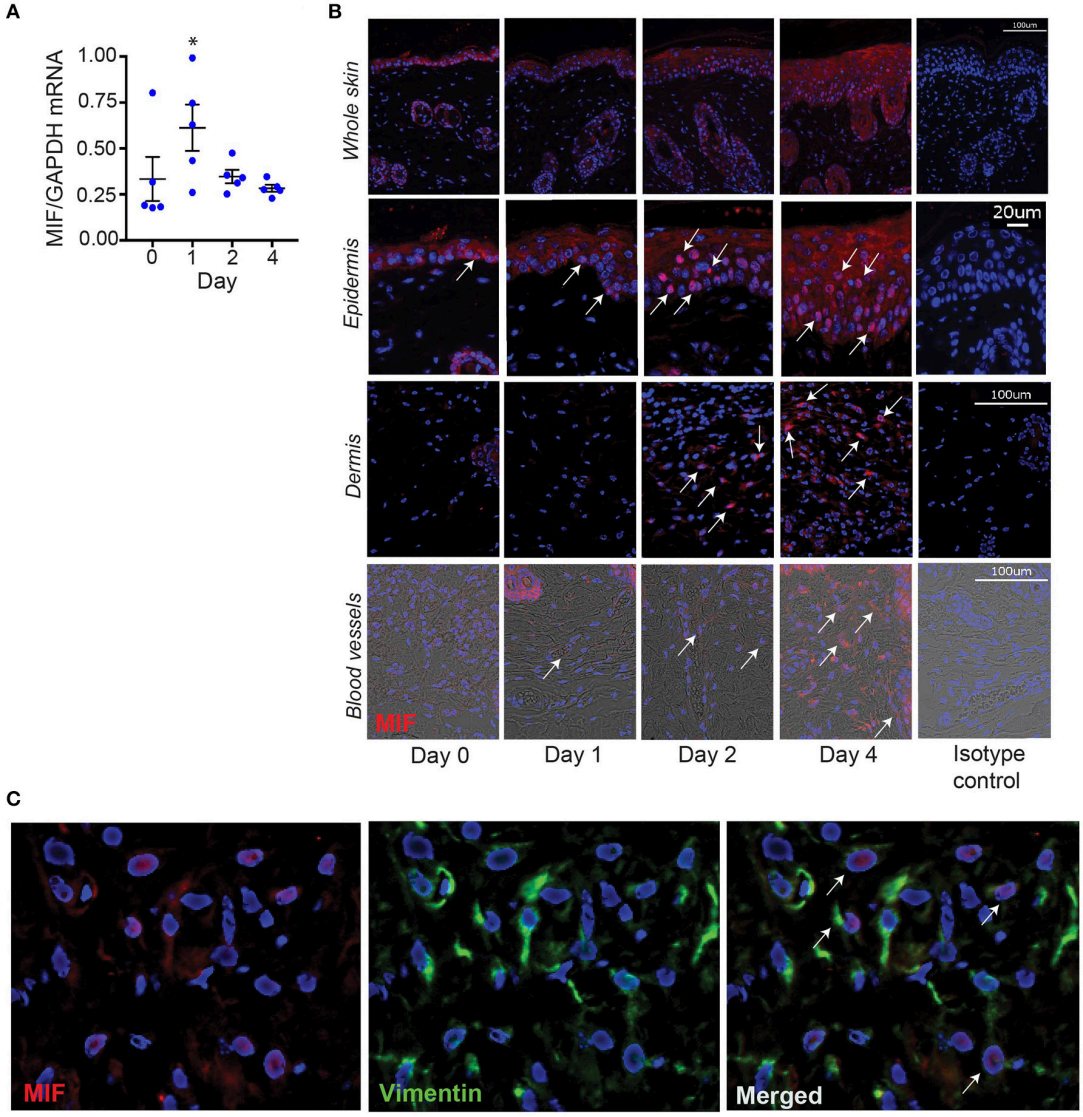
MIF expression is increased in the skin in IIPD. The dynamic of MIF expression was determined in Aldara™ treated skin of WT mice on **(A)** mRNA level and **(B)** protein level. The latter is illustrated by representative pictures of immunofluorescence stainings for MIF showing overviews in the first panel and pictures focused on the epidermis, dermis, and dermal blood vessels. Arrows indicate examples of spots of MIF protein expression. Scale bars represent 100 or 20 μm, as stated in the pictures on the far-right column. **(C)** Lesional skin of a representative wild-type mouse harvested on day 4 in the IIPD mouse model was stained for MIF (red) and the fibroblast marker vimentin (green). Overlaying MIF and vimentin expression revealed that in the dermis MIF is predominantly expressed in vimentin-positive cells. White arrows in the magnification of the overlay indicate MIF/vimentin double-positive cells. Results in **(A)** were analyzed for statistical significance by Kruskal-Wallis test with Dunn's multiple comparison test. ^*^p < 0.05 compared to expression on day 0. Results are presented as mean ± SEM (*n* = 5 mice/time point). One representative of two experiments is shown.

To identify potential cellular sources of MIF in IIPD, we assayed MIF expression at the mRNA and protein level in psoriasiform dermatitis by qPCR and immunofluorescence staining, respectively. In accord with previous reports (5–8), MIF was constitutively expressed in the skin, predominantly in the basal keratinocyte layer (Figures 4A,B). Upon Aldara™ application, MIF mRNA levels in the skin increased by 2.5-fold within 24 hrs and remained elevated until the end of the experiment on day 4 (Figure 4A). Immunofluorescence staining showed that MIF was markedly induced in keratinocytes, but also, although to a much lesser extent, in the dermis (Figure 4B). In the latter, MIF was predominantly expressed in vascular endothelial cells (Figure 4B) or in dermal fibroblast, which were identified by their expression of vimentin (Figure 4C). MIF serum levels did not change upon induction of IIPD (results not shown), indicating that the release of MIF in response to Aldara™ is rather restricted to the skin.

**Figure 5:**
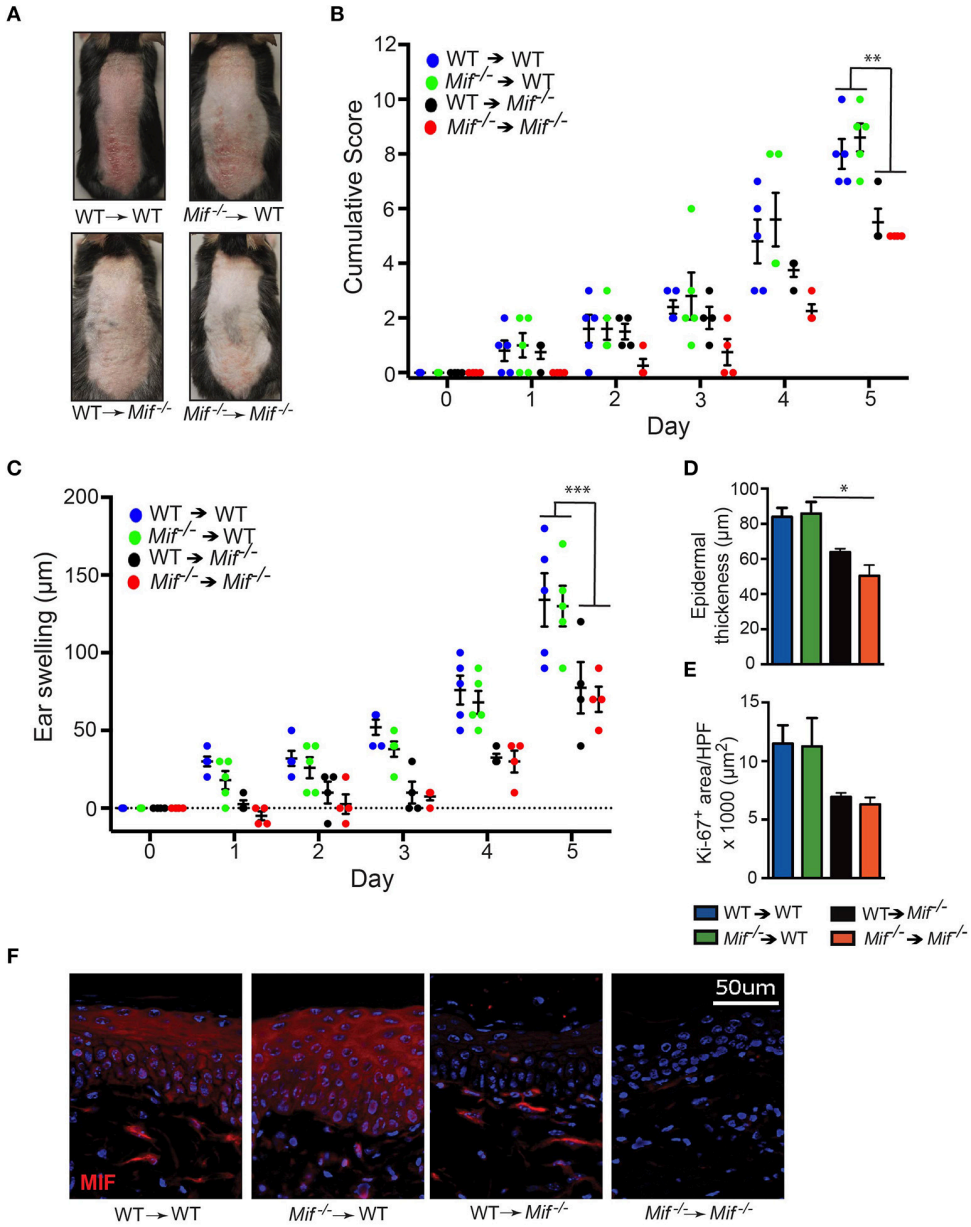
MIF in radiosensitive cells is sufficient to drive IIPD. IIPD was examined in all reciprocal bone marrow chimera between wild-type and *Mif*^−/−^ mice. **(A)** Representative clinical pictures of back skin on day of the experiment. **(B)** Cumulative score of IIPD disease activity on back skin and **(C)** ear swelling day 0 through day 5. **(D)** Epidermal thickness and **(E)** Ki-67^+^ area per HPF in back skin on day 5. **(F)** MIF protein expression on day 5. Results are presented as mean ± SEM (4–5 = mice/group). Results in **(B)** and **(C)** were analyzed by two-way ANOVA with Holm-Sidak's multiple comparison test, results in **(D)** and **(E)** were tested by Kruskal-Wallis test with Dunn's multiple comparison test. ^*^p < 0.05; ^**^p < 0.01; ^***^p < 0.001.

To corroborate that MIF derived from skin resident, radioresistant cells, such as keratinocytes, fibroblasts, and vascular endothelial cells, is the major source of pathogenically relevant MIF, we conducted one experiment generating all reciprocal bone marrow chimera of wild-type and *Mif*^−/−^ mice, precisely *Mif*^−/−^ → *wild-type*, and *wild-type* → *Mif*^−/−^ chimera as well as their controls *wild-type* → *wild-type*, and *Mif*^−/−^ → Mif^−/−^ mice. Eight weeks after bone marrow engraftment all four groups were subjected to the IIPD model and skin inflammation was evaluated clinically by scoring back skin inflammation and assessing ear swelling. Disease severity was equivalent in *wild-type* → *wild-type* and *Mif*^−/−^ → *wild-type* chimera, but was decreased in *wild-type* → *Mif*^−/−^ and *Mif*^−/−^ → *Mif*^−/−^ chimera when compared to the first two groups, indicating specifically that MIF derived from radioresistant cells aggravates psoriasiform dermatitis (Figures 5A–C). This contribution of radioresistant cell-derived MIF for skin inflammation was also reflected on the histopathological level, where epidermal hyperplasia was reduced in *Mif*^−/−^ → *wild-type* and *Mif*^−/−^ → *Mif*^−/−^ chimera (Figures 5D,E). Most MIF expressing cells in inflamed skin were radioresistant, skin resident cells (Figure 5F).

### *Mif* deficiency does not affect CCL2 or IL-17A expression levels in the IIPD model

**Figure 6:**
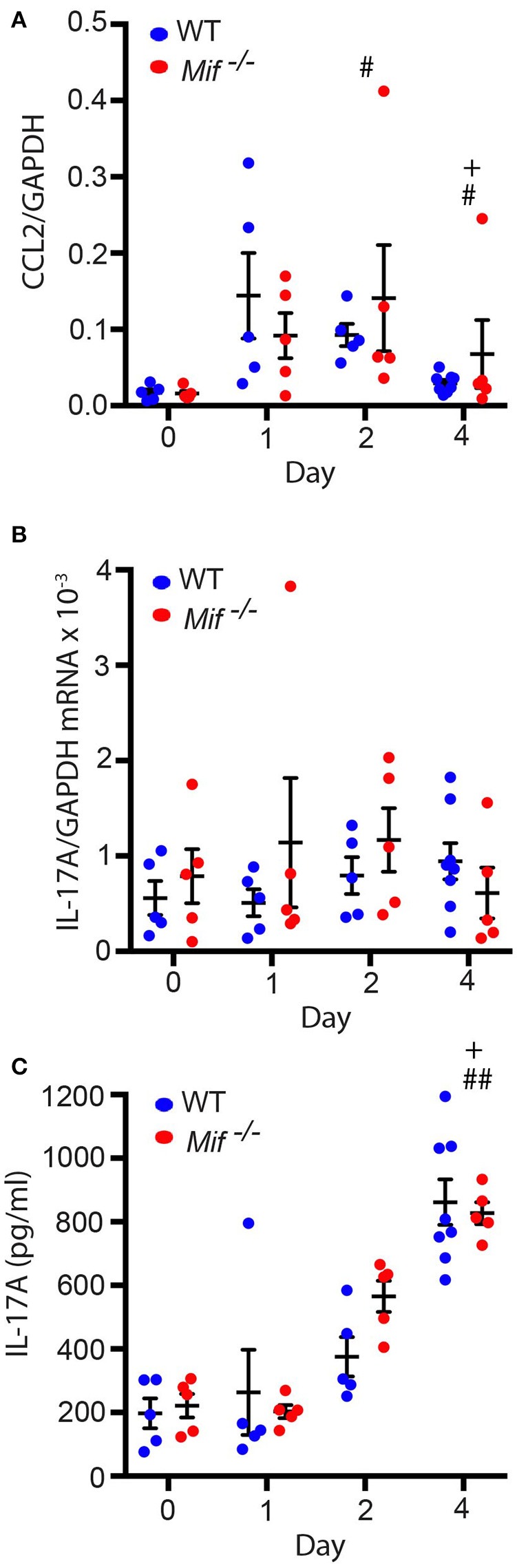
Mif deficiency does not affect CCL2 or IL-17A levels in the IIPD model. Comparison of wild-type and *Mif* ^−/−^ mice in the IIPD model over time with respect to **(A)** CCL2 mRNA and **(B)** IL-17A mRNA expression in lesional skin and to **(C)** serum levels of IL-17A. Results are presented as mean ± SEM and were compared by Kruskal-Wallis test with Dunn's multiple comparison test (*n* = 5–8 mice/group). ^#^*p* < 0.05; ^##^*p* < 0.01 WT mice day 0 *vs*. WT mice day indicated; ^+^*p* < 0.05 *Mif* ^−/−^ mice day 0 *vs. Mif* ^−/−^ mice day indicated. One representative of two independent experiments is shown.

As it had been previously reported that MIF can regulate monocyte recruitment into tissues by inducing the monocyte-active chemokine CCL2 (MCP-1) (10), we next addressed the effect of MIF deficiency on CCL2 expression in the skin by profiling CCL2 mRNA levels in lesional skin of wild-type and *Mif*^−/−^ mice by qPCR throughout the course of IIPD. Significant amounts of CCL2 mRNA were present in the skin of both wild-type and *Mif*^−/−^ mice already in healthy skin and increased upon treatment with Aldara™. There was no significant difference in its expression levels between wild-type and *Mif*^−/−^ mice (Figure 6A).

MIF was previously also reported to reinforce the release of IL-17A from lymph node cells (31), and IL-17 has been implicated in the pathogenesis of IIPD (15). This prompted us to also examine the effect of MIF deficiency on IL-17A levels in the IIPD model. IL-17A mRNA was equally expressed in the skin of wild-type and *Mif*^−/−^ mice and only showed tendency toward a slight increase in its expression levels upon Aldara™ treatment (Figure 6B). IL-17A serum levels, in contrast, were significantly increased upon treatment with Aldara™, but they did not differ between the two strains either (Figure 6C).

### MIF contributes to psoriasiform dermatitis in the IL-23-induced dermatitis model

**Figure 7:**
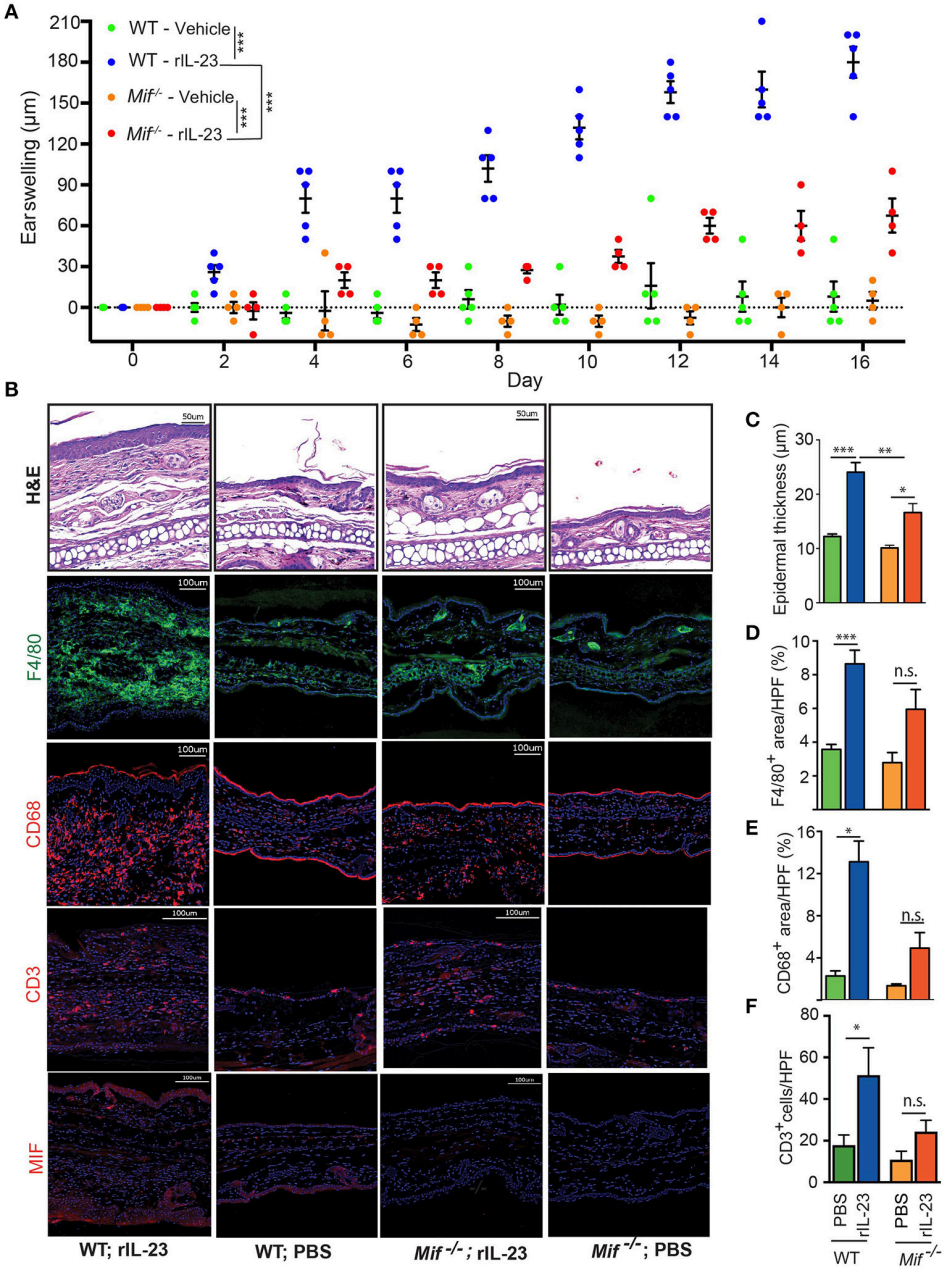
MIF is crucial for IL-23-induced dermatitis. Comparison of WT and *Mif* ^−/−^ mice in IL-23-induced dermatitis. **(A)** Time course of ear swelling. **(B)** Representatives pictures of H&E, F4/80, CD68, CD3, and MIF stainings of recombinant IL-23- or PBS-treated skin harvested on day 16. Quantification of **(C)** epidermal thickness, **(D)** F4/80^+^ areas/HPF, **(E)** CD68^+^ areas/HPF, and **(F)** number of CD3^+^ cells/HPF on day 16. In **(A)**, results were compared by two-way ANOVA with Holm-Sidak's multiple comparison test, in **(C)** – (F) by Kruskal-Wallis test with Dunn's multiple comparison test. ^*^p < 0.05; ^**^p < 0.01; ^***^p < 0.01 (*n* = 5 mice/group). One representative of two experiments is shown. Scale bars represent 50 or 100 μm, as indicated in the pictures.

With the IL-23/IL-17 pathway most critical in both IIPD and human psoriasis (18, 32, 33), we set out to investigate the role of MIF specifically in this pathway. To this end, we scrutinized the role of MIF in the IL-23-induced dermatitis mouse model. In this model, psoriasiform dermatitis is induced by intradermal application of recombinant IL-23 (14). 0.5 μg recombinant IL-23 or its vehicle control (1% BSA in PBS) were injected i.d. into the dorsal aspect of the ears of wild-type and *Mif*^−/−^ mice every other day. The severity of dermatitis was assessed by determining ear swelling. In wild-type mice, IL-23 precipitated psoriasiform dermatitis around the injection site and induced significant ear swelling. By contrast, in *Mif*^−/−^ mice, dermatitis was almost completely abrogated (Figure 7A). This difference was also reflected on the histopathological level (Figure 7B). Thus, in wild-type mice, IL-23 treatment induced significant epidermal hyperproliferation and epidermal thickening, which were significantly reduced in *Mif*^−/−^ mice (Figure 7C). Like Aldara™, recombinant IL-23 increased the expression of MIF in the skin (Figure 7B). Furthermore, it induced the recruitment of monocytes and T cells into the dermis of wild-type mice. This recruitment was attenuated in *Mif*^−/−^ mice, where the recruitment of both monocytes and T cells into the skin did not reach statistically significant levels (Figures 7B–F).

## Discussion

We have addressed the pathogenic significance of MIF in psoriasis using the IIPD and the IL-23-induced dermatitis mouse models. In both models, MIF was abundant in lesional skin, and its absence significantly reduced disease severity. The most pronounced histopathological difference *Mif*^−/−^mice exhibited in comparison to wild-type mice in both models was a reduction in keratinocyte hyperproliferation. This indicates that MIF directly or indirectly promotes the uncontrolled proliferation of keratinocytes in psoriasiform dermatitis. Notably, in support of the notion of a direct effect of MIF on keratinocytes proliferation, it was previously reported to induce the proliferation of primary keratinocytes *in vitro* (34). With MIF highly expressed in keratinocytes, this proliferative effect may be due to auto- and paracrine effects of MIF in the epithelium.

The infiltration of the dermis by diverse immune cell lines is another hallmark of psoriasis. In line with previous studies, in both, the IIPD and the IL-23-induced dermatitis model, the emergence psoriasiform dermatitis was associated with marked infiltration of the dermis with monocyte-derived cells and T cells as well as, though to a lesser extent, with neutrophils. Previous reports demonstrated that in both models particularly the recruitment of T cells and monocytes into the skin is crucial for the course of disease (23, 35–38). We found differential effects of MIF deficiency on the recruitment of these cell lines as well as some differences between the two models. Thus, MIF deficiency reduced the peak of monocyte-derived cell infiltration in the dermis in the course of the IIPD model, but had no detectable effect on T cell infiltration in this model. As a corollary, MIF may promote psoriasiform dermatitis in the IIPD model by facilitating monocyte recruitment into the dermis. Furthermore, our results suggest that this effect on the recruitment of monocytes is not mediated *via* an enhancement of CCL2 levels by MIF. The notion of MIF as regulator of monocyte recruitment into the skin is in line with a recent study highlighting dermal fibroblast- and keratinocyte-derived MIF as a pivotal regulator of the number of monocyte-derived cells in the dermis (8).

In the IL-23-induced dermatitis model, we did not find a statistically significant difference in monocyte-derived cells or in T cells in the dermal infiltrate between IL-23 treated wild-type and *Mif*^−/−^ mice, but the numbers of both tended to be reduced. This suggests that MIF may have a modulatory, promoting effect on the recruitment of both into the dermis.

Conclusively elucidating the relative contribution of the suggested modes of action of MIF to psoriasiform dermatitis and further distinguishing similarities and differences of the role of MIF in the two models requires future studies employing cell type-specific CD74 deficient mice, thus, distinguishing the effects of MIF on individual cell types.

The IIPD and IL-23-induced dermatitis mouse models both reflect major clinical, histopathological, and molecular features of human plaque psoriasis. They are, therefore, widely used to investigate the pathogenesis of this disease and to develop new therapeutic strategies (16). The high expression of MIF and its cellular pattern in lesional skin, found here, are additional features these models have in common with human plaque psoriasis. This further corroborates the validity of these models for the human disease. Considering the high level and cellular expression pattern of MIF in psoriatic skin lesions in humans, it is conceivable that MIF may play a similar role in the pathogenesis of the human disease. This highlights MIF as possible drug target in the treatment of this disease. A therapeutic strategy inhibiting MIF would be most intriguing because MIF is paradoxically induced by glucocorticoids and counteracts their immunosuppressive actions (39). Hence, MIF inhibitors may synergize with topical glucocorticoids, which are still the mainstay in the treatment of acute flares of plaque psoriasis, thus, sparing glucocorticoid toxicity. Furthermore, with MIF implicated in atherogenesis (40, 41), which is purportedly aggravated by plaque psoriasis, MIF may be a common pathogenic pathway of both diseases. Thus, its therapeutic inhibition may be beneficial for both the skin and the cardiovascular disease in psoriasis patients.

The physiological function of MIF in skin immunology may be to jumpstart host defense because, like Aldara™, a wide array of pathogens, including bacteria, fungi, and viruses, activate TLR7 and/or inflammasomes. In line with this notion, it has lately been demonstrated that intracellular MIF is involved in the activation of the NLRP3 inflammasome in monocytes/macrophages (42, 43). It is therefore worth mentioning here that the NLRP3 inflammasome was previously excluded to play a role in IIPD (44). The immune response of the skin against pathogens also often includes epidermal hyperproliferation, such as in nummular eczema and tinea, which are caused by streptococci and dermatophytes, respectively. MIF is poised for this putative central role in skin immunity because, unlike other cytokines and chemokines with the notable exception of IL-1α/β, MIF is constitutively expressed in the skin and can be immediately released. This enables MIF to rapidly initiate immune responses against invading pathogens. Through this function in host defense, MIF may promote the pathogenesis of psoriasis and the emergence of psoriatic lesions, which often emerge in response to exogenous microbial stimuli.

## Author contributions

SB, LL, SM, DR, MD, and HB conducted the experiments and analyzed the results. RB and CS planned the study and evaluated the results. CS wrote the paper. DZ edited the paper and evaluated the results.

### Conflict of interest statement

The authors declare that the research was conducted in the absence of any commercial or financial relationships that could be construed as a potential conflict of interest.
