# Supplementary material for: Macrophage Migration Inhibitory Factor (MIF) Drives Murine Psoriasiform Dermatitis
Source: Front Immunol. 2018 Oct 2;9:2262. doi: 10.3389/fimmu.2018.02262 (PMC6176003; doi:10.3389/fimmu.2018.02262)
Supplement: Supplementary file 1 [file Table_1.DOCX]

| **Gene** | **Forward primer 5’🡪3’** | **Reverse primer 5’🡪3’** |
| --- | --- | --- |
| *Ccl2* | GGCTCAGCCAGATGCAGTTA | GGTGATCCTCTTGTAGCTCTCC |
| *Gapdh* | AGGTCGGTGTGAACGGATTTG | TGTAGACCATGTAGTTGAGGTCA |
| *Il17a* | TCAGCGTGTCCAAACACTGAG | CGCCAAGGGAGTTAAAGACTT |
| *Mif* | GCCAGAGGGGTTTCTGTCG | GTTCGTGCCGCTAAAAGTCA |

**Table S1 – Sequences of primers used in this study**
