# Supplementary material for: Macrophage Migration Inhibitory Factor (MIF) Drives Murine Psoriasiform Dermatitis
Source: Front Immunol. 2018 Oct 2;9:2262. doi: 10.3389/fimmu.2018.02262 (PMC6176003; doi:10.3389/fimmu.2018.02262)
Supplement: Supplementary file 2 [file Table_2.DOCX]

Table S2 – Commercially availabel primary and secondary antibodies used in this study

| **Primary antibody** | **Vendor** | **Secondary antibody** | **Vendor** | **Fluorophor** | **Vendor** |
| --- | --- | --- | --- | --- | --- |
| Rat anti-mouse Ki-67 | Biolegend, UK | Biotinylated goat anti-rat IgG | Thermo Fisher Scientific GmbH, Germany | Streptavidine conjugated DyLight 594 | Thermo Fisher Scientific GmbH, Germany |
| Polyclonal rabbit anti-mouse CD31 | Abcam, UK | Donkey anti-rabbit IgG AlexaFluor 594 | Biolegend, UK | n/a | n/a |
| Biotinylated rat anti-mouse Ly6G IgG2a | Biolegend, UK | n/a | n/a | Streptavidine conjugated DyLight 488 | Thermo Fisher Scientific GmbH, Germany |
| Biotinylated Armenian Hamster anti-mouse CD3 | Biolegend, UK | n/a | n/a | Streptavidine conjugated DyLight 594 | Thermo Fisher Scientific GmbH, Germany |
| Rat anti-mouse F4/80 IgG2b | AbD Serotec, UK | Biotinylated goat anti-rat IgG | Thermo Fisher Scientific GmbH, Germany | Streptavidine conjugated DyLight 488 | Thermo Fisher Scientific GmbH, Germany |
| Alexa Fluor^®^ 594 rat anti-mouse CD68 IgG2a | Biolegend, UK | n/a | n/a | n/a | n/a |
| Goat anti-vimentin | Santa Cruz, USA | Alexa Fluor 488 conjugated Donkey anti-goat | Jackson Immuno Research,  USA | n/a | n/a |
